# Authors’ Response to Peer Reviews of “Applications of Indocyanine Green in Breast Cancer for Sentinel Lymph Node Mapping: Protocol for a Scoping Review”

**DOI:** 10.2196/68769

**Published:** 2025-01-06

**Authors:** Feryal Kurdi, Yahya Kurdi, Igor Vladimirovich Reshetov

**Affiliations:** 1 Department of Oncology, Radiotherapy and Plastic and Reconstructive Surgery, Sechenov University, Bolshaya Pirogovskaya, 6c1, Moscow, 119021, Russian Federation, 7 9013488810

**Keywords:** indocyanine green, ICG, sentinel lymph node, breast cancer, breast, fluorescence, axillary lymph node mapping, NIR, surgical planning, near-infrared

*This is the authors’ response to peer-review reports for “Applications of Indocyanine Green in Breast Cancer for Sentinel Lymph Node Mapping: Protocol for a Scoping Review.”*

## Round 1 Review

### Anonymous [1]

#### General Comments

*This paper [* *2* *] summarized the application value and existing problems of indocyanine green (ICG) in sentinel lymph node (SLN) biopsy of early breast cancer, which has positive significance for improving the accuracy of clinical SLN detection. This study has certain clinical value.*

Response: Thank you for your thoughtful comments and feedback on our paper. Below are my responses to your points.

#### Specific Comments

##### Major Comments

*1. Due to the high hardware requirements for the clinical application of ICG, the number of relevant studies in the search is relatively small. It is hoped that the author can search the recent, relevant literature to improve the credibility of this review.*

Response: This paper is a protocol for a scoping review, serving as a roadmap for the search strategy and inclusion criteria that we will follow. As such, it outlines our plan rather than reporting the outcomes of the literature search. As noted in Multimedia Appendix 1, we will conduct a comprehensive search across multiple databases to ensure the inclusion of all relevant, recent studies.

*2. It is hoped that the author will analyze and compare the advantages and disadvantages of ICG and traditional SLN biopsy methods, so as to guide clinicians to adopt appropriate methods for appropriate patients.*

Response: As indicated in Multimedia Appendix 1, this comparison is a core objective of our review. We hope these clarifications address your concerns.

## Abbreviations

ICG: indocyanine green
SLN: sentinel lymph node
